# The Probiotic Attributes and Anti-pseudorabies Virus Evaluation of *Lactobacillus* Isolates

**DOI:** 10.3389/fvets.2022.902052

**Published:** 2022-06-20

**Authors:** Ming-Fan Yang, Wei Yan, Yan Li, Shuai-Qi Li, Hong-Ying Chen, Qing-Qiang Yin, Xiao-Wei Dang, Hong-Ying Zhang

**Affiliations:** ^1^Zhengzhou Key Laboratory for Pig Disease Prevention and Control, College of Veterinary Medicine, Henan Agricultural University, Zhengzhou, China; ^2^Henan Delin Biological Products Co., Ltd., Zhengzhou, China

**Keywords:** *Lactobacillus plantarum*, *Lactobacillus casei*, probiotic potential, pseudorabies virus, antiviral

## Abstract

The emergence of pseudorabies virus (PRV) variants brings serious harm to the swine industry, and its effective treatments are limited at present. As one of the probiotics, the *Lactobacillus* species have beneficial characteristics of regulating the balance of intestinal flora, inhibiting the growth of pathogenic bacteria and viruses' proliferation, and improving self-immunity. In this study, *Lactobacillus plantarum* HN-11 and *Lactobacillus casei* HN-12 were selected and identified through morphology observation, Gram stain microscopy, 16S rRNA sequencing analysis, and specific amplification of the recA gene and pheS gene. All tested isolates exhibited rapid adaptation to the different conditions, excellent acid, and bile tolerance, and sensitivity to *Salmonella, Staphylococcus aureus*, and *Escherichia coli*. The antibiotic susceptibility assay displayed the isolates sensitive to most antibiotics and resistant to Lincomycin and Norfloxacin. Moreover, the supernatants of HN-11 and HN-12 inhibited PRV proliferation in ST cells. The results of animal experiments showed that supplementing the challenged mice with the supernatants of *Lactobacillus* isolates in advance delayed the course of the disease. PRV was detected in the heart, liver, spleen, lung, kidney, and brain tissues of dead mice in the test groups, and its copies in the lungs were significantly decreased compared with the control mice (*P* < 0.05). These findings proved the advantages of *L. plantarum* and *L. casei* as potential probiotic cultures, which could provide a basis for its application in microecological preparations and functional formulations.

## Introduction

Probiotics are defined as live microorganisms by WHO and exert a beneficial effect on the body if given in sufficient amounts (1). A fact that probiotics play an important role in regulating intestinal microecology had been determined (2). And the disturbance of intestinal microecology is related to the decline of the body's production performance, which in turn causes a variety of diseases. As the most widely used and extensively studied probiotic, *Lactobacillus* spp, such as *Lactobacillus plantarum, Lactobacillus casei*, and *Lactobacillus bifidum*, is known to prevent or treat gastrointestinal disorders in both humans and animals (3). A previous study confirmed that weaned piglets fed with diet supplements of *Lactobacillus* had a lower rate of diarrhea and mortality than those with the control diets by regulating intestinal flora disorders (4). In addition, *Lactobacillus* species produce lactic acids, bacteriocins, and other metabolites to achieve the purpose of antibacterial, and many *Lactobacillus* preparations play an essential role in enhancing immunity (5). Remarkably, the antiviral effect of *Lactobacillus* and its metabolites has attracted attention globally in recent years (6).

As an epidemic swine pathogen, pseudorabies virus (PRV) causes significant economic losses to the global swine industry and is characterized by acute and high fever and neurological symptoms in piglets, abortions and stillbirths in sows, and digestive system diseases, such as vomiting, diarrhea, necrotizing enteritis, in adult pigs (7). Pseudorabies (PR) caused by PRV is a serious infectious disease, with mortality of nearly 100% in piglets (8). The swine is the natural host for PRV, and the virus can also infect most mammals, including cattle, rodents, and dogs (9). *Lactobacillus* isolates and their metabolites have been confirmed to exhibit antiviral activity against several human and animal viruses (10). However, there is no relevant research and report on *Lactobacillus* inhibiting PRV. Therefore, this study aimed to evaluate the probiotic properties of the *L. plantarum* and *L. casei* isolated from fermented silage and also to determine their antiviral activity against PRV, which provided the basis for its application in animal husbandry production.

## Materials and Methods

### Samples, Cells, Viruses, and Animals

The corn silage samples used for the isolation of *Lactobacillus* in this study were collected from the silage cellars of a large-scale sheep farm in Henan Province, China.

Swine testicle (ST) cells were previously preserved in our laboratory and were cultured in Dulbecco's modified Eagle's medium (DMEM, Solarbio, Beijing, China) containing 10% (v/v) fetal bovine serum and 100 U mL^−1^ penicillin/streptomycin at 37°C and 5% CO_2_ for 48 h. PRV was previously stored in our laboratory. The preparation of viral inoculum was as follows: The virus suspension was adsorbed on the ST monolayer cells for 1.5 h and washed three times with PBS and then added DMEM containing 2% fetal bovine serum to culture under 37°C and 5% CO_2_ until the appearance of 80% CPE. Finally, the cells along with the culture medium were frozen and thawed thrice, and centrifuged for the supernatant. Female mice (3–4 weeks old, Kunming, SPF) were purchased from Henan Experimental Animal Center and allowed to eat and drink freely throughout the experiment.

### Isolation and Genotypic Characterization

An amount of 10 g of each sample was cut into small pieces, suspended in PBS and the supernatant was plated on Man Rogosa Sharp (MRS) agar. The suspected bacteria colonies were randomly selected to striate on plates containing MRS agar and incubated at 37°C for 48 h, and then the colonies were purified and further evaluated for Gram reaction and morphology. To identify the isolates, the total genomic DNA of each isolate was extracted by the Rapid Bacterial Genomic DNA Isolation kit (Sangon Biotech, Shanghai, China) and amplified *via* PCR using a pair of primers (27F: 5′ - AGAGTTTGATCCTGGCTCAG-3′ and 1492R: 5′-TACGGCTACCTTGTTACGACT-3′). The PCR has carried out with the following program: denaturation at 95°C for 5 min, 35 cycles of 95, 52, and 72°C/45 s, and extension at 72°C for 10 min. After the amplification, 6 μL PCR products were separated by electrophoresis in a 1.2% (w/v) agarose gel, visualized by Gel Imaging System (Sigma, USA), and sequenced by TSINGKE company (Beijing, China). Finally, the sequencing results were compared with the known sequences in the database of NCBI using the BLAST program, and the phylogenetic tree was constructed using the neighbor-joining method with MEGA 7 software.

To identify the PCR products of the recA and pheS gene, the genomic DNA of the isolates HN-11 and HN-12 was amplified by specific primer pairs (pF: 5′ - CTATTTTTCGGTTGGTTGGTCG-3′, pR: 5′-TTGGCTGATGCACGGAAAG-3′, cF: 5′ -ATGGATCTTCAAACCAAACTTGAAC-3′ and cR: 5′ -TTAACCCTCCTGGCTGAATTGC-3′), respectively. The PCR program was carried out above, and products were detected by 1.2% agarose gel electrophoresis.

### Growth Curves

For growth curves, bacteria were grown on agar plates and a single colony was transferred to a 10 ml MRS broth and incubated overnight, then 6 mL fresh cultures were inoculated into 300 ml of MRS broth and incubated at 37°C for 18–36 h. The growth was monitored by measuring optical density (OD) at 600 nm for every 2 h interval, and pH was measured at the same time points.

### Acid and Bile Tolerance

The acid and bile tolerance of isolates were checked according to the methods described previously with minor modifications (11). Acid tolerance was examined in MRS broth and adjusted to a final pH of 2.5 using 1N HCl. Specifically, 1 mL overnight cultured *Lactobacillus* isolates were inoculated into 9 ml MRS broth previously adjusted to pH 2.5 and cultured anaerobically at 37°C for 3 h. Bile tolerance was analyzed as above that 1 ml of overnight cultured isolates were inoculated into 9 ml MRS broth with 0.3% (w/v) oxgall bile salt (Sigma) and incubated at 37°C for 8 h. Then the plate counting method was used to measure the viable bacteria after incubation and the initial. Finally, the survival rate (%) of acid and bile tolerance was calculated as the percentage of the number of viable bacteria grown on MRS agar after incubation (N_1_, lg CFU/mL) and the initial number of viable bacteria (N_0_, lg CFU/mL) according to the formula: survival rate (%) = N_1_/ N_0_ × 100%.

### Antibacterial Activity

The antibacterial activity of *Lactobacillus* isolates on indicator bacteria was evaluated by agar well diffusion assay as described previously (12). Indicator bacteria, such as *E. coli, S. aureus*, and *Salmonella*, were prepared in a bacterial suspension with a final concentration of 10^7^ CFU ml^−1^ and inoculated into Luria-Bertani (LB) agar. The *Lactobacillus* suspension was injected into a well with a diameter of 5 mm which was punched in the LB agar plates as the experimental groups, and the wells were filled with MRS liquid medium as control groups. The antibacterial activity was measured as the halos of the inhibition zone after incubation at 37°C for 48 h. The inhibitory effect was assessed by the width of the inhibition halos and ranked as highly sensitive (>15 mm), Moderately sensitive (10–15 mm), low sensitive (5–10 mm), and not sensitive (5 mm).

### Antibiotic Susceptibility Assay

The isolates were evaluated for susceptibility to Penicillin, Cefamezin, Gentamicin, Clarithromycin, Lincomycin, Norfloxacin, and Rifampicin by the disc diffusion method using commercially available antibiotic disks. Bacteria cultures were diluted to suitable turbidity (0.6 OD at 600 nm) and spread on MRS agar. The antibiotic disks were placed at a suitable distance with sterile forceps. Then the plates were incubated at 37°C for 24–48 h. Zone of inhibition (mm) was measured, and the results were described as sensitive (S) or resistant (R) according to National Committee for Clinical Laboratory Standards (NCCLS).

### Antiviral Assay *in vitro*

Isolate supernatants were tested for PRV inhibition *in vitro* in ST cells. Ten-fold serial dilutions of the viral inoculum were prepared, and each dilution was inoculated into 8 wells. After incubation for 72 h, CPE was observed and the viral titers in the wells were calculated as the 50% tissue culture infective dose (TCID_50_/100 μL) using the Karber method. The *Lactobacillus* isolates were cultured anaerobically in MRS broth at 37°C for 16 h, and supernatants collected by centrifugation at 12,000 rpm for 5 min were adjusted with PBS to 10^8^ CFU ml^−1^ and filter-sterilized 0.22-μm syringe filter. The cytotoxicity of isolates supernatants on ST cells were tested *in vitro* using a Cell Counting Kit-8 (CCK-8) assay. Different concentrations of supernatants were inoculated into a 96-well monolayer of ST cells, and the wells of blank control and virus control were also maintained. After the plate was incubated at 37°C with 5% CO_2_ for 48–72 h, 10 μL of a CCK-8 solution was added, and OD at 450 nm was measured. The relative cell viability was counted as a percentage of that of the control based on the mean OD. ST Monolayers in 6-well plates were inoculated with three different safe and effective concentrations of the supernatants of *Lactobacillus* isolates after which it was infected with 100TCID_50_ of PRV inoculum and allowed to absorb for 2 h as test groups, and quadruplicate wells of the virus control and blank control were also established. After the virus wells had developed 80% CPE, the plates were freeze-thawed thrice at −80°C. The mixtures were harvested and centrifuged to prepare the supernatants for the extraction of viral DNA. The PRV copies were determined using the fluorescence quantitative PCR (qPCR), and the primers and methods were the same as described previously (13).

### Animal Treatments

After the adaptation period, a total of 48 Kunming mice in this part were randomly divided into the HN-11 group, HN-12 group, blank control group, and positive control group (*n* = 4), each group was set for three repetitions. And from day 8 to day 17, mice in the experiment groups and control groups were forced to accept the 1 mL supernatants of HN-11 and HN-12 at 10^8^ CFU mL^−1^ and an equal volume of PBS, respectively. Afterward, mice in the HN-11, HN-12, and positive control groups were subcutaneously injected with 250 μL of PRV inoculum with a proliferation titer of 10^5.17^TCID_50_/100 μL according to previous experiments.

### Sample Collection

The health status of the mice was closely observed and recorded, including diet, mental state, and morbidity. All dead mice were immediately dissected and their tissues, including heart, liver, spleen, lung, kidney, and brain, were collected. The viral DNA was extracted, and antigens were determined by qPCR as above.

### Statistical Analysis

Experimental results were analyzed for statistical significance using GraphPad Prism (GraphPad, San Diego, USA). Independent Student *t*-test analysis was performed. *P* < 0.05 was defined as the statistical significance level. The values were expressed as the mean ± SD.

## Results

### Isolation and Genotypic Characterization of Isolates

**Figure 1:**
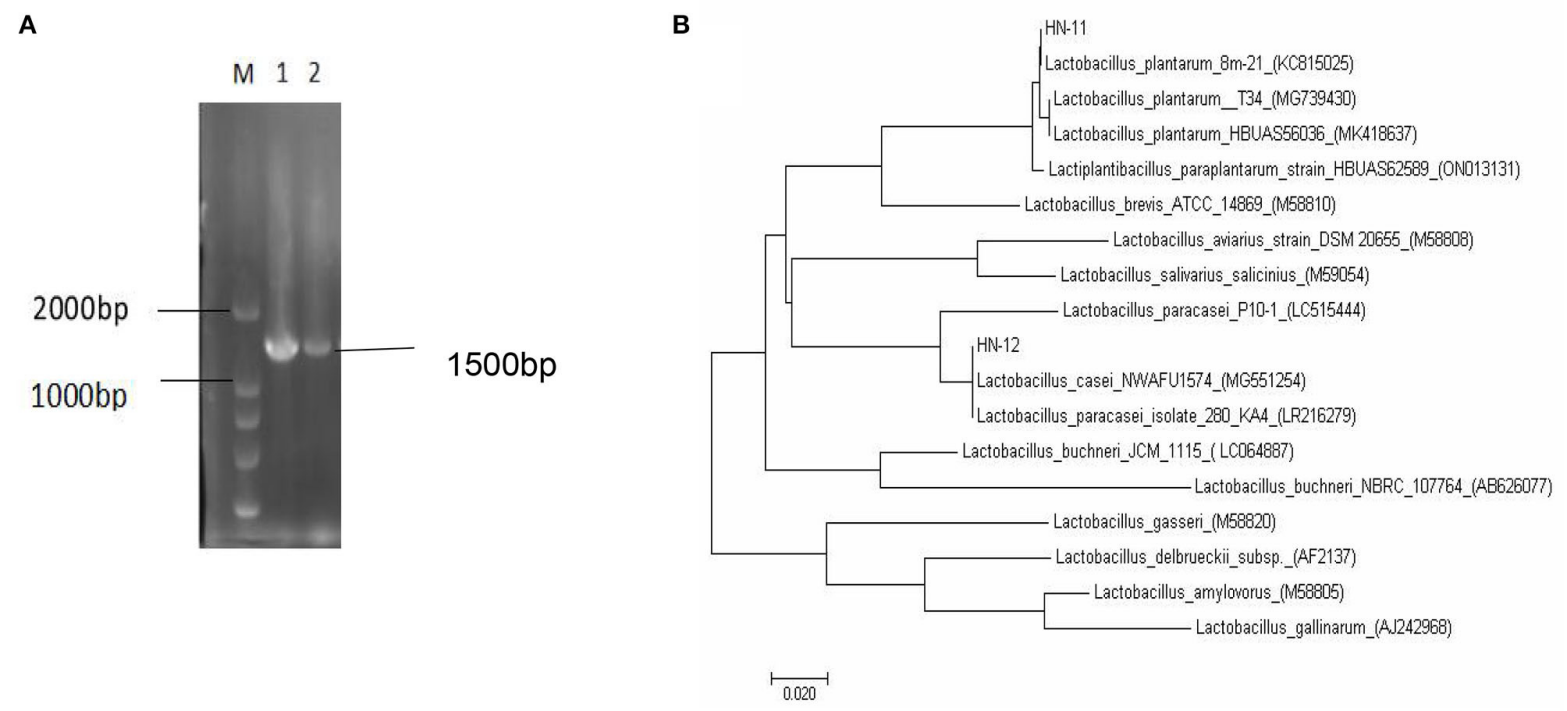
The 16S rRNA gene sequence homology analysis of *Lactobacillus* isolates. **(A)** Amplified PCR products of about 1,500 bp; M. DNA marker DL2000; 1. isolate HN-11; 2. isolate HN-12. **(B)** Phylogenetic tree based on the 16S rRNA gene sequence of *Lactobacillus* isolates showing the relationship of isolates to other strains already present in GenBank. And the tree was rebuilt by the neighbor-joining method.

A total of 18 gram-positive colonies with typical *Lactobacillus* morphology, white in color and round in shape were selected and inoculated into MRS broth. The 16S rRNA gene sequence was matched with the reference sequence in the NCBI database to identify the organism. Blast analysis showed a similarity percentage ≥97% of homology to *Lactobacillus* species. The phylogenetic tree reconstructed by the neighbor-joining method reveals a phylogenetic relationship between the sequences of the 16S rRNA gene of the obtained strains (Figure 1).

**Figure 2:**
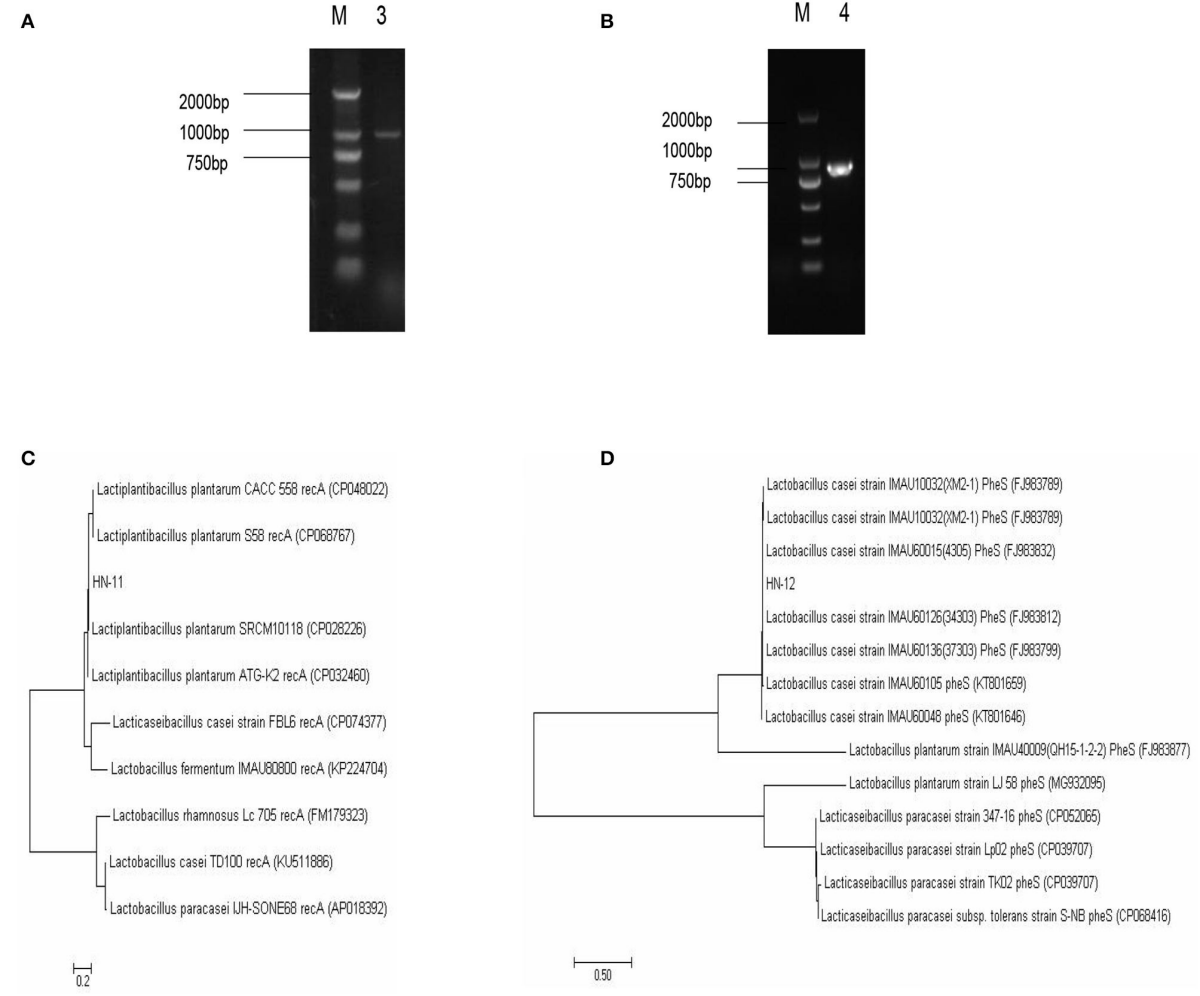
The identification of *Lactobacillus* isolates by specific gene sequences. **(A,B)** Amplified PCR product of about 1,000 bp; M. DNA marker DL2000; 3. the specific amplification products of recA gene of *Lactobacillus plantarum* HN-11; 4. the specific amplification products of pheS gene of *Lactobacillus casei* HN-12. The phylogenetic tree based on the recA gene **(C)** and pheS gene **(D)** of *Lactobacillus* isolates shows the relationship of isolates to other strains already present in GenBank. And the tree was rebuilt by the neighbor-joining method.

The homology of the l6S rRNA of *Lactobacillus* species is too high to distinguish. Therefore, a specific pair of primers (pF and pR) was designed based on the conserved genes recA of *L. plantarum*, and the predicted amplified fragment length was 1,143 bp. Moreover, we also obtained specific primer pairs (cF and cR) based on the pheS gene sequences of the *L. casei* group, and the predicted amplified fragment length was 1,047 bp. The amplification products of the recA gene of the isolate HN-11 and the amplification product of the pheS gene of HN-12 were similar to the predicted results (Figures 2A,B). The target amplified sequence was analyzed by BLAST and the phylogenetic trees were reconstructed by the neighbor-joining method (Figures 2C,D). The result shows that the isolate HN-11 had high homology with *Lactiplantibacillus plantarum* strain SRCM101187 (CP028226), and isolate HN-12 had high homology with *L. casei* strain IMAU60126 (34303) (FJ983812). Above all, the isolates HN-11 and HN-12 were identified as *L. plantarum* and *L. casei*, respectively.

### Growth Curves

**Figure 3:**
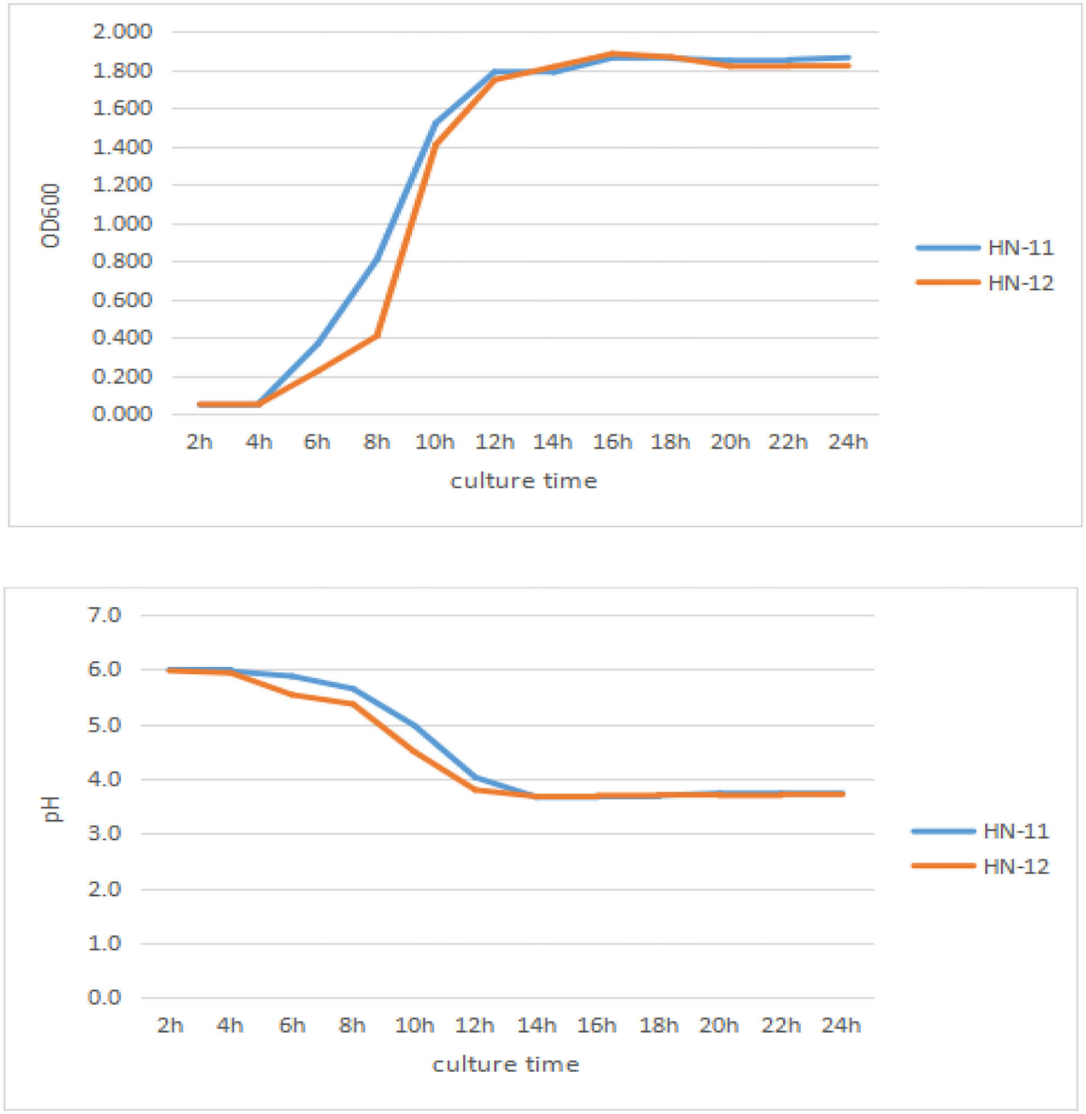
Growth curves and acid production of the three isolates. The culture time was taken as abscissa, and OD_600nm_ value and pH value were taken as ordinate. HN-11 is labeled in blue, and HN-12 is labeled in orange.

As can be seen in Figure 3, the lag phase of three isolates lasted 4 h and three isolates reached the stationary phase after 16 h incubation, with a final OD_600nm_ of 1.7 or so. Good adaptation capability could help bacteria adapt to different environments, which might improve probiotic function in the gut. And the pH value of the isolates rapidly dropped to 3.72 at 14 h and last for a long time.

### Acid and Bile Tolerance

Table 1 shows that the isolates showed a survival rate of more than 95% after incubation for 3 h at a pH of 2.5. They also survived in 0.3% bile and showed more than 90% of the survival rate after 8 h incubation. Despite a reduction of the viable count (lg CFU/ml) after incubation, the number of viable bacteria was still 10^7^ CFU/ml above. A study has shown that the minimum number of viable bacteria for LAB to play a probiotic effect is 10^6^ CFU/ml (14).

**Table 1:** Acid and bile tolerance of *Lactobacillus*^a^.

**Strains**	**Acid tolerance (pH: 2.5, 3 h)**			**Bile tolerance (0.3%, 8 h)**		
	**0 h (lg CFU/mL)**	**3 h (lg CFU/mL)**	**Survival rate (%)**	**0 h (lg CFU/mL)**	**8 h (lg CFU/mL)**	**Survival rate (%)**
HN-11	7.78 ± 0.02	7.64 ± 0.05	98.20	8.08 ± 0.03	7.49 ± 0.03	92.70
HN-12	7.63 ± 0.05	7.36 ± 0.05	96.46	7.93 ± 0.04	7.28 ± 0.04	91.80

a *Each value is the mean ± SD of data*.

### Antibacterial Activity

The isolates were evaluated for antibacterial activity against pathogenic bacteria by agar well diffusion assay (Table 2). In the assay, the strains HN-11 and HN-12 showed highly (>15 mm) sensitivity to *Salmonella* and *S. aureus*. On the other hand, they showed low sensitivity (6–10 mm) to *E. coli*.

**Table 2:** Antibacterial activity of *Lactobacillus* isolates determined by agar well diffusion assay^1^.

**Strains**	**Inhibition hale (mm)**		
	***Salmonella***	***Staphylococcus aureus***	***Escherichia coli***
HN-11	18.73 ± 0.73	17.00 ± 0.16	7.47 ± 0.25
HN-12	17.00 ± 0.16	16.03 ± 0.21	7.80 ± 0.33

1 *Results are shown as mean ± SD. Experiments were carried out thrice independently*.

### Antibiotic Susceptibility Assay

The susceptibility patterns of three *Lactobacillus* isolates against seven antibiotics are shown in Table 3. Both the isolates were sensitive to Cefamezin, Penicillin, Gentamicin, Clarithromycin, and Rifampicin but resistant to Lincomycin and Norfloxacin.

**Table 3:** Antibiotic susceptibility test of *Lactobacillus* isolates.

**Antimicrobials**	**Strains**	
	**HN-11**	**HN-12**
Cefamezin (30 ug)	S	S
Penicillin (10 U)	S	S
Gentamicin (10 ug)	S	S
Clarithromycin (15 ug)	S	S
Lincomycin (2 ug)	R	R
Norfloxacin (10 ug)	R	R
Rifampicin (5 ug)	S	S

*S, sensitive; R, resistant*.

### Antiviral Assay *in vitro*

**Figure 4:**
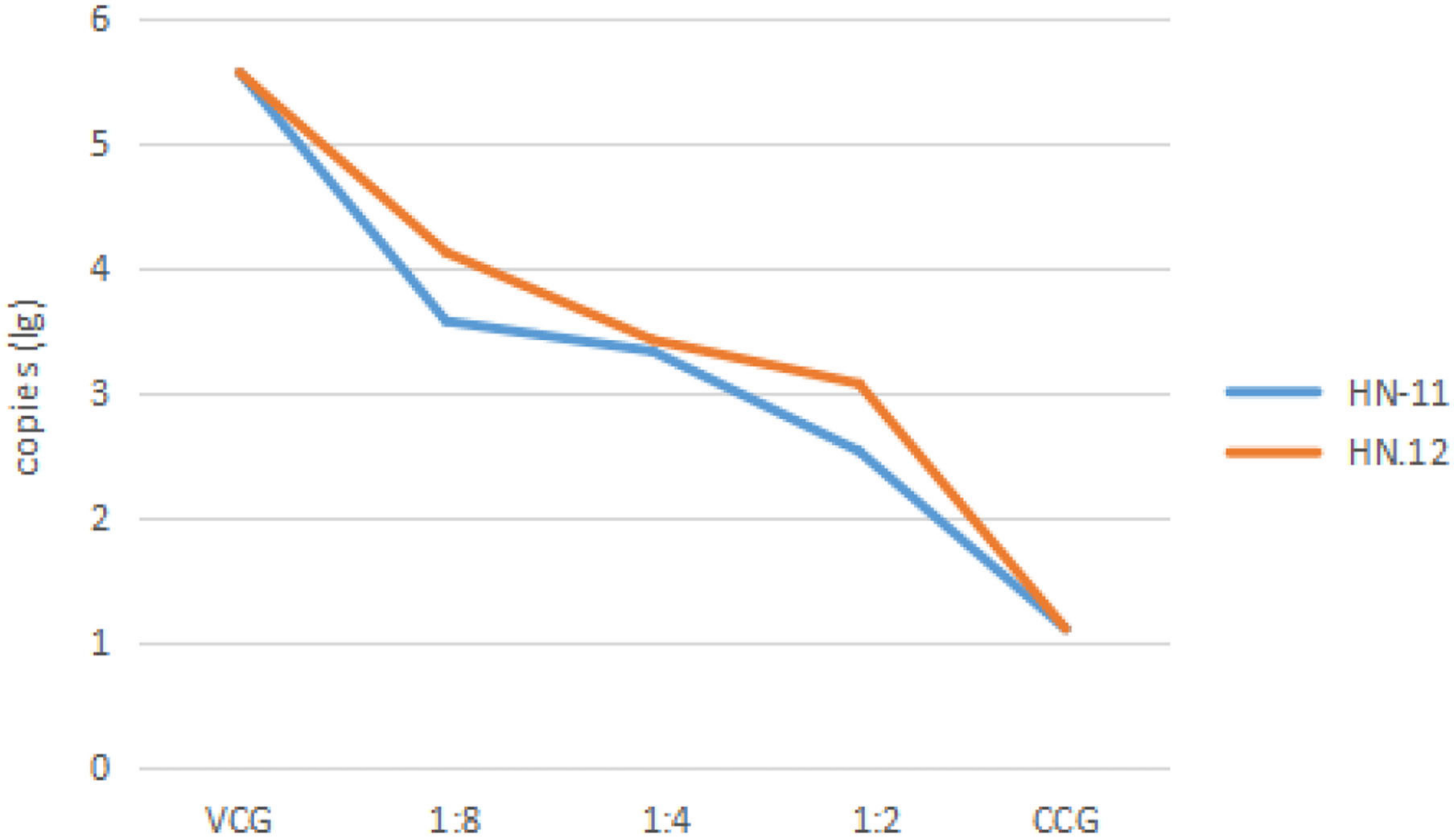
The copies of the virus after treatment with cell-free supernatants of isolates in different dilutions. The dilution of 1:8, 1:4, and 1:2 of cell-free supernatants of isolates, cell control groups, and virus control groups are taken as abscissa, and the copies (lg) are taken as ordinate. VCG, the virus control groups; CCG, the cell control groups. HN-11 is labeled in blue, and HN-12 is labeled in orange.

The virulence of the PRV inoculum was measured on ST cells as 10^5.17^ TCID_50_/100 μL. And the maximum non-toxic dose of the supernatant of isolates was the dilution factor of 1:2. Therefore, the dilution gradient of *Lactobacillus* supernatant is 1:2, 1:4, and 1:8 in the next antiviral effect test. The DNA of the test groups and control groups was amplified, and the amplification results were analyzed as Figure 4 that the copies of the virus in the test groups were lower than that in the virus control groups, and the copies decreased with the decrease of dilution factor of the supernatant, respectively.

### Antiviral Assay *in vivo*

**Figure 5:**
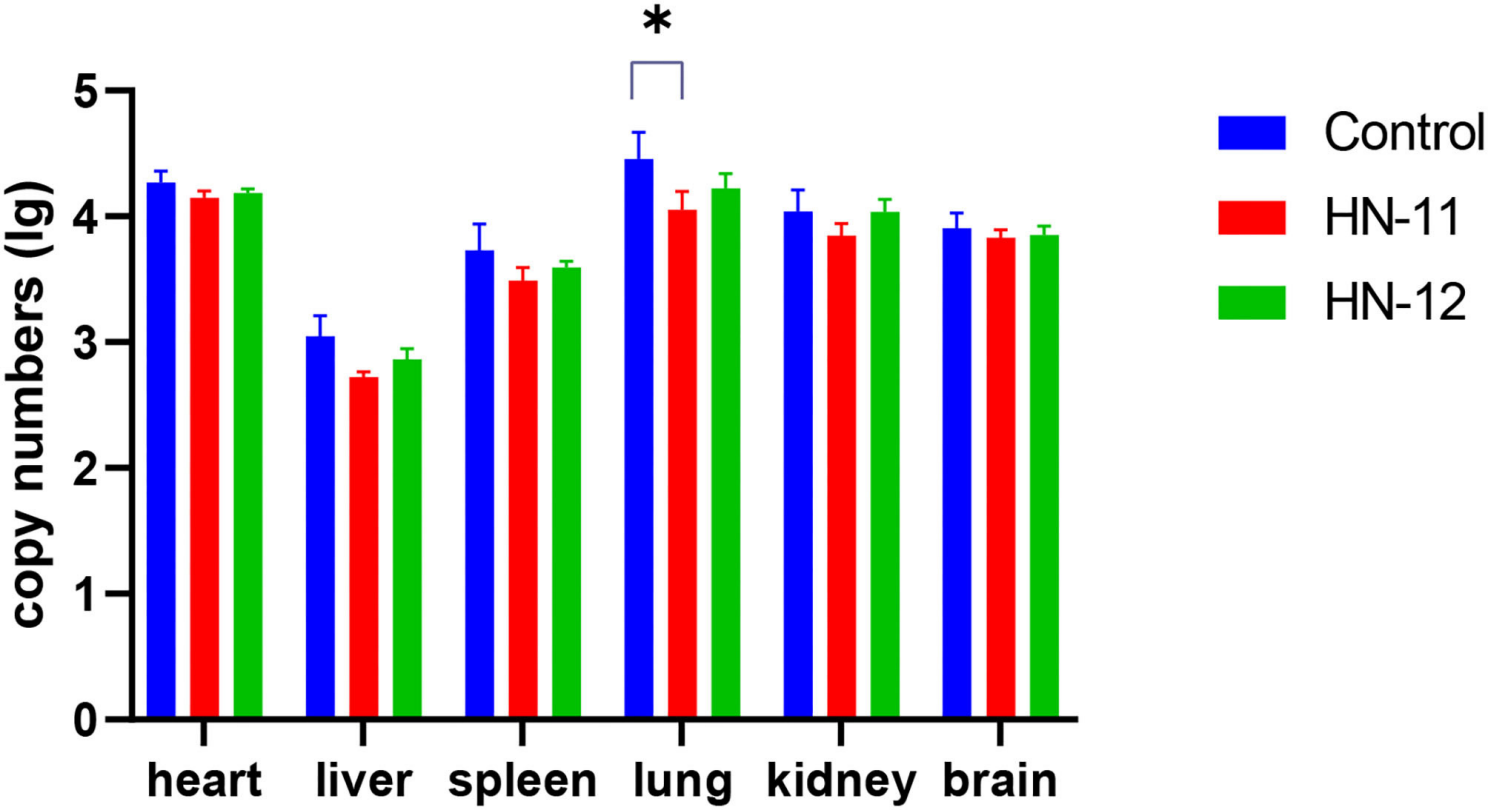
The copies of PRV in mice tissues. The heart, liver, spleen, lung, kidney, and brain tissues of the positive control group, HN-11 group, and HN-12 group are taken as abscissa, and the copies (lg) are taken as ordinate (*P < 0.05).

In this experiment, the mortality of mice after 7 days of challenge with PRV was shown in Table 4 that the mice in the control group died on the 3 and 4 days after PRV challenge and no mice died in the blank control group. Some mice in the experimental group showed depression, decreased diet, severe pruritus, and accelerated breathing before death. The results showed that the protective effect of *Lactobacillus* isolates on the mice artificially infected with PRV inoculum was not significant, but it delayed the course of the disease. PRV was detected in the heart, liver, spleen, lung, kidney, and brain tissues of dead mice in the experimental group, and the viral load in tissues in the *Lactobacillus*-supplemented groups was lower than in the blank control group, but the difference was not significant (*p* > 0.05) (Figure 5). However, the viral load in lung tissues of the mice supplemented with isolate HN-11 was significantly reduced compared with the control mice (*P* < 0.05).

**Table 4:** Mortality results of experiments mice.

**Date**	**The mortality of mice**			
	**Positive control**	**Blank control**	**HN-11**	**HN-12**
	**group**	**group**	**group**	**group**
1 dpi	0	0	0	0
2 dpi	0	0	0	0
3 dpi	6	0	4	6
4 dpi	6	0	4	3
5 dpi	0	0	2	2
6 dpi	0	0	2	0
7 dpi	0	0	0	1
Remaining mice	0	12	0	0

## Discussion

The health-promoting property of probiotics motivated the present study to isolate novel *Lactobacillus* with potential probiotic attributes. Any bacteria to be considered as potential probiotics should show high resistance to acidic conditions and bile salts (15). According to our study, both isolates had viability percentages of more than 90% against bile and higher tolerance to an acid environment, which is an important guarantee to ensure that *Lactobacillus* pass through the gastrointestinal barrier in the form of live bacteria. A total of 30 strains of *Lactobacillus* isolated from infants' feces were 58–97% survival in acid at pH 2.5 and 50–94% survival in 0.3% bile salts (3), which is similar to the results of this study. The efficacy of antimicrobial activity against pathogens exhibited by both isolates is beneficial for maintaining the intestinal flora balance, which is an important property of ideal probiotics. Śliżewska et al. (16) suggested several *Lactobacillus* strains as probiotic candidates for the development of functional feed due to the probiotic properties they exhibit, such as high survival rate and antagonistic activity, against pathogens. *L. plantarum* with excellent probiotic properties had been proved to relieve diarrhea caused by enterotoxigenic *E. coli* (ETEC) (17). Xu et al. (18) reported the antibacterial activity of *L. casei* NA-2 against *Bacillus cereus, S. aureus, S. typhimurium*, and *E. coli* O157:H7, together with its potential antibacterial properties of exopolysaccharide (EPS). An increasing data has shown the EPS from *Lactobacillus* exerts antibacterial activities by inhibiting biofilm formation and dispersing of various bacteria, such as *S. aureus* (19), *E. coli* (16), *Listeria monocytogenes, S. typhimurium*, and *P. aeruginosa* (20). Further studies are required to determine the EPS product secreted by the present strains to differentiate them from previous strains.

Through TANJA, it was confirmed the antiviral activity of probiotics and their metabolites in *in vitro* cell model for the first time in 2007 (21). Since then, the cell infection model has been widely used in probiotic antiviral tests. Previous reports found that *L. plantarum* metabolites can inhibit the adsorption of transmissible gastroenteritis virus (TGEV) on ST cells (22). Moreover, both *L. plantarum* metabolites and *L. plantarum* exopolysaccharides showed a good inhibitory effect on porcine epidemic diarrhea virus (PEDV) (23) and rotaviruses (RV) (24), and the treatments that exerted the excellent effects have been repeatedly proved. This could be due to the complex composition of metabolites, which blocks virus adsorption, alleviates inflammatory responses, and induces immunity (14). We herein observed that PRV inhibition on the ST model by isolated supernatant was concentration-dependent. Currently, there is a lack of animal test models, but the results of this test showed that *Lactobacillus* isolates were indeed impossible to independently complete the task of antiviral on mice model, but probiotics can still be supplemented clinically to assist vaccines or antiviral drugs to achieve a better effect. As previously reported, *L. acidophilus* had significant immuno-potentiating effects and was suggested as a safe oral adjuvant for RV vaccines in neonates gnotobiotic pigs (25). And further studies are required to explore the antiviral effect of specific substances of probiotic metabolites.

*Lactobacillus* isolates (HN-11 and HN-12) had been confirmed to have excellent probiotic properties and the obtained data showed the supernatants all make a certain contribution in inhibiting the proliferation of PRV *in vitro* and *in vivo*. In an era defined by severe antibiotic resistance in bacterial pathogens, probiotic feeding may represent a potential effect on reducing antimicrobial use and restoring animal health.

## Data Availability Statement

The original contributions presented in the study are included in the article/supplementary material, further inquiries can be directed to the corresponding author/s.

## Ethics Statement

The animal study was reviewed and approved by the Henan Agriculture University Animal Care and Use Committee [license number SCXK (Henan) 2013-0001].

## Author Contributions

M-FY and WY have made substantial contributions to the conception, design of the work, acquisition, and analysis. YL and S-QL carried out interpretation of data. H-YC discussed and prepared the final report. Q-QY and X-WD contributed to the drafting of the work. H-YZ has completed the revision of the entire paper and provided financial support. All authors have read and approved the final manuscript.

## Funding

This study was supported by the Project for National Natural Science Foundation of China-Research on Bidirectional Immunomodulatory Effects of Radix Isatidis polysaccharide on PRRSV-infected 3D4/21 cells (31972731).

## Conflict of Interest

X-WD is employed by Henan Delin Biological Products Co., Ltd. The remaining authors declare that the research was conducted in the absence of any commercial or financial relationships that could be construed as a potential conflict of interest.

## Publisher's Note

All claims expressed in this article are solely those of the authors and do not necessarily represent those of their affiliated organizations, or those of the publisher, the editors and the reviewers. Any product that may be evaluated in this article, or claim that may be made by its manufacturer, is not guaranteed or endorsed by the publisher.
